# A comparative analysis of exome capture

**DOI:** 10.1186/gb-2011-12-9-r97

**Published:** 2011-09-29

**Authors:** Jennifer S Parla, Ivan Iossifov, Ian Grabill, Mona S Spector, Melissa Kramer, W Richard McCombie

**Affiliations:** 1 Cold Spring Harbor Laboratory, 1 Bungtown Road, Cold Spring Harbor, New York 11724, USA

## Abstract

**Background:**

Human exome resequencing using commercial target capture kits has been and is being used for sequencing large numbers of individuals to search for variants associated with various human diseases. We rigorously evaluated the capabilities of two solution exome capture kits. These analyses help clarify the strengths and limitations of those data as well as systematically identify variables that should be considered in the use of those data.

**Results:**

Each exome kit performed well at capturing the targets they were designed to capture, which mainly corresponds to the consensus coding sequences (CCDS) annotations of the human genome. In addition, based on their respective targets, each capture kit coupled with high coverage Illumina sequencing produced highly accurate nucleotide calls. However, other databases, such as the Reference Sequence collection (RefSeq), define the exome more broadly, and so not surprisingly, the exome kits did not capture these additional regions.

**Conclusions:**

Commercial exome capture kits provide a very efficient way to sequence select areas of the genome at very high accuracy. Here we provide the data to help guide critical analyses of sequencing data derived from these products.

## Background

Targeted sequencing of large portions of the genome with next generation technology [1-4] has become a powerful approach for identifying human variation associated with disease [5-7]. The ultimate goal of targeted resequencing is to accurately and cost effectively identify these variants, which requires obtaining adequate and uniform sequencing depth across the target. The release of commercial capture reagents from both NimbleGen and Agilent that target human exons for resequencing (exome sequencing) has greatly accelerated the utilization of this strategy. The solution-based exome capture kits manufactured by both companies are of particular importance because they are more easily adaptable to a high-throughput workflow and, further, do not require an investment in array-processing equipment or careful training of personnel on array handling. As a result of the availability of these reagents and the success of the approach, a large number of such projects have been undertaken, some of them quite large in scope.

As with many competitive commercial products, there have been updates and improvements to the original versions of the NimbleGen and Agilent solution exome capture kits that include a shift to the latest human genome assembly (hg19; GRCh37) and coverage of more coding regions of the human genome. However, significant resources have been spent on the original exome capture kits (both array and solution) and a vast amount of data has been generated from the original kits. We therefore analyzed two version 1 exome capture products and evaluated their performance and also compared them against the scope of whole genome sequencing to provide the community with the information necessary to evaluate their own and others' published data. Additionally, our investigation of factors that influence capture performance should be applicable to the solution capture process irrespective of the actual genomic regions targeted.

While exome sequencing, with a requirement of 20-fold less raw sequence data compared to whole genome sequencing [5], is attractive, it was clear that based on the number of regions targeted by the initial commercial reagents compared to the number of annotated exons in the human genome that not all of the coding regions of the genome were targeted. Moreover, our qualitative analyses of our previous exon capture results indicated a marked unevenness of capture from one region to another in exome capture based on such factors as exon size and guanine-cytosine (GC) context [3].

To gain a more thorough understanding of the strengths and weaknesses of an exome sequencing approach, comparative analyses were done between two commercial capture reagents and between exome capture and high coverage whole genome sequencing. The results show that the commercial capture methods are roughly comparable to each other and capture most of the human exons that are targeted by their probe sets (as described by Consensus Coding Sequences (CCDS) annotations). However, they do miss a noteworthy percentage of the annotated human exons described in CCDS annotations when compared to high coverage, whole-genome sequencing. The limitations of the two commercial exome capture kits we evaluated are even more apparent when analyzed in the context of coverage of the more comprehensive RefSeq annotations [8,9], which are efficiently covered by whole genome sequencing.

## Results

### Characteristics of commercially available solution exome capture kits

Two exome capture platforms were evaluated: NimbleGen SeqCap EZ Exome Library SR [10] and Agilent SureSelect Human All Exon Kit [11]. These two commercial platforms are designed to provide efficient capture of human exons in solution, they require smaller amounts of input DNA compared to the previous generation of array-based hybridization techniques, and they support scalable and efficient sample processing workflows. Both platforms are designed to target well-annotated and cross-validated sequences of the human hg18 (NCBI36.1) exome, based on the June 2008 version of CCDS [12]. However, because the probes used for each kit were designed using algorithms specific to the particular platform, the two kits target different subsets of the approximately 27.5 Mb CCDS. The Agilent SureSelect system uses 120-base RNA probes to target 165,637 genomic features that comprise approximately 37.6 Mb of the human genome, whereas the NimbleGen EZ Exome system uses variable length DNA probes to target 175,278 genomic features covering approximately 26.2 Mb of the genome.

Each kit targets the majority of the approximately 27.5-Mb CCDS database: NimbleGen 89.8% and Agilent 98.3%. However, they each cover somewhat different regions of the genome. We found by comparing the 37.6 Mb Agilent target bases to the 26.2 Mb NimbleGen target bases that 67.6% of the Agilent target bases are included in the NimbleGen targets and 97.0% of the NimbleGen target bases are included in the Agilent targets.

### Solution exome capture with the 1000 Genomes Project trio pilot samples

Six samples from two trios (mother, father, and daughter) that had been sequenced in the high-coverage trio pilot of the 1000 Genomes Project [13] were used: one trio is from the European ancestry in Utah, USA population (CEU) and one trio from the Yoruba in Ibadan, Nigeria population (YRI). Table 1 shows the specific sample identifiers. We obtained purified genomic DNA from cell lines maintained at Coriell Cell Repositories in Coriell Institute for Medical Research (Camden, NJ, USA) and carried out multiple exome capture experiments using both the NimbleGen and Agilent solution-based exome capture products. Using the NimbleGen kit we performed one independent capture for each of the CEU trio samples, two independent captures for the YRI father sample, and four independent captures for the YRI mother and YRI daughter samples. Using the Agilent kit we performed four independent captures for the YRI mother and YRI daughter samples (Table 1).

**Table 1:** Human DNA samples and exome captures used in this study

				Number of captures	
				
Our ID	Population	HapMap ID	Family member	NimbleGen	Agilent
CEU-M	CEU	NA12892	Mother	1	0
CEU-F	CEU	NA12891	Father	1	0
CEU-D	CEU	NA12878	Daughter	1	0
YRI-M	YRI	NA19238	Mother	4	4
YRI-F	YRI	NA19239	Father	2	0
YRI-D	YRI	NA19240	Daughter	4	4

The human genomic DNA samples used for this study were chosen to match those analyzed by the trio pilot of the 1000 Genomes Pilot [13]. The sources of the genetic material consist of a European ancestry in Utah, USA population (CEU) trio and a Yoruba in Ibadan, Nigeria population (YRI) trio, with each trio composed of a mother, a father, and a daughter. The table reviews the exome captures performed with each sample. For DNA samples from which multiple exome captures were performed, independent libraries were prepared from each DNA sample in order to support independent exome captures. Exome captures were performed with either the SeqCap EZ Exome Library SR (NimbleGen) or the SureSelect Human All Exon Kit (Agilent), which were designed to capture well-annotated protein-coding regions of the human hg18 (NCBI36.1) assembly. Following exome capture, each captured library was sequenced using paired-end 76-cycle chemistry, in one flowcell lane on the Genome Analyzer_IIx _instrument (Illumina).

Each captured library was sequenced in a single lane of a Genome Analyzer_IIx _instrument (Illumina, Inc.) using paired-end 76-cycle chemistry. The pass-filter Illumina sequence data were analyzed for capture performance and genetic variants using a custom-designed bioinformatics workflow (see Materials and methods). This workflow imposed stringent filtering parameters to ensure that the data used downstream for variant detection were of high quality and did not have anomalous characteristics. To evaluate capture performance, the pipeline performed the following steps: (1) filter out bases in a given read that match the Illumina PCR oligos used to generate the final library; (2) map the reads to the human hg18 reference using Burrows-Wheeler Aligner (BWA) [14] and only retain read pairs with a maximal mapping quality of 60 [15] and with constituent reads spanning a maximum of 1,000 bp and oriented towards each other; (3) remove replicate read pairs that map to identical genomic coordinates; and (4) remove reads that do not map to platform-specific probe coordinates. The last step was integrated into the pipeline in order to allow rigorous evaluation and comparison of the targeting capabilities of the capture kits, since non-specific reads generated from the capture workflow were likely to be inconsistent between capture experiments (data not shown). Given that most of our sequence data were retained following each filtering step, we conclude that most of our exome capture data were of good quality to begin with. A full bioinformatics report of the results of our exome capture data analysis is provided in Additional file 1.

### Exome coverage differs between two solution capture platforms

We first examined the exome coverage with respect to the intended targets of the two platforms. These targets were determined based on the information provided by NimbleGen and Agilent. There is an important difference in the way the two companies define and provide their targets. NimbleGen provides an 'intended target' that comprises the regions (exons) for which they expected to be able to design probes for, whereas Agilent only provides their 'intended target' based on their final probe design. This difference in 'intended target' definition leads to a substantial difference in the intended target sizes: 26.2 Mb for NimbleGen and 37.6 Mb for Agilent. On the other hand, the genomic space covered by the exome probes is more comparable between the two companies, which is likely due to various methodological similarities in hybridization probe design. The NimbleGen probes span 33.9 Mb of genomic space, and the Agilent probes span 37.6 Mb of genomic space.

**Figure 1:**
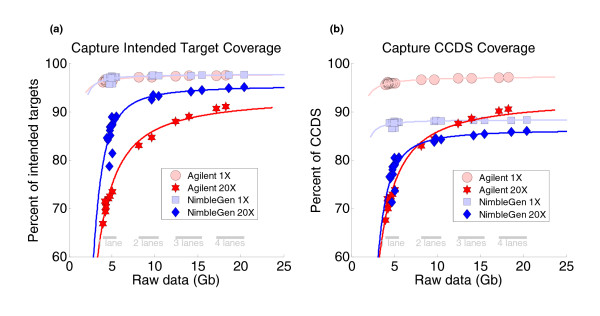
**Targeting efficiency and capability varied between commercially available exome capture kits**. **(a) **The intended targets of the NimbleGen and Agilent exome kits were 26,227,295 bp and 37,640,396 bp, respectively. Both exome kits captured similarly high amounts (up to about 97%) of their intended targets at 1× depth or greater, but the NimbleGen kit was able to reach saturation of target coverage at 20× depth more efficiently than the Agilent kit. The NimbleGen exome kit required less raw data to provide sufficient coverage of the exome and to support confident genotype analysis. **(b) **Both exome kits were designed to target exons based on the June 2008 version of CCDS, which consisted of 27,515,053 bp of genomic space. Notably, the NimbleGen target was smaller than the CCDS, while the Agilent target was larger than the CCDS. Based on 1× depth sequence coverage, the Agilent exome kit captured more of the CCDS than the NimbleGen exome kit (97% covered by Agilent versus 88% covered by NimbleGen), but the NimbleGen kit was more efficient at capturing the regions of the CCDS it had the capability to capture.

**Figure 2:**
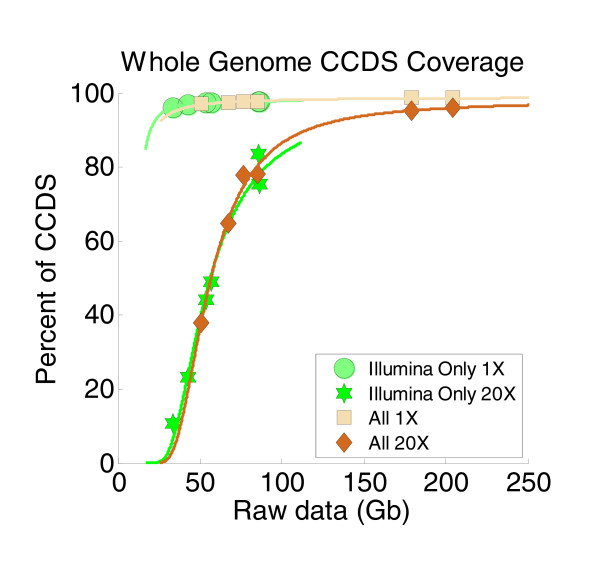
**With enough raw data, whole genome sequencing could achieve almost complete coverage of the CCDS (intended target of the exome capture kits)**. Approximately 98% of CCDS was covered at 1× or greater and approximately 94% covered at 20× or greater from the more deeply sequenced daughter samples. To generate this plot depicting the relationship between CCDS coverage depth and raw sequence data input, we imposed a coverage model based on two assumptions: that CCDS coverage depth should match genome coverage depth, and that genome size (3 Gb) times the desired coverage depth is the amount of raw sequence data (in gigabases) necessary to achieve such depth. Illumina Only, only the alignment files from Illumina sequence data were used; All, alignment files from Illumina, 454, and SOLiD sequence data were used.

**Figure 3:**
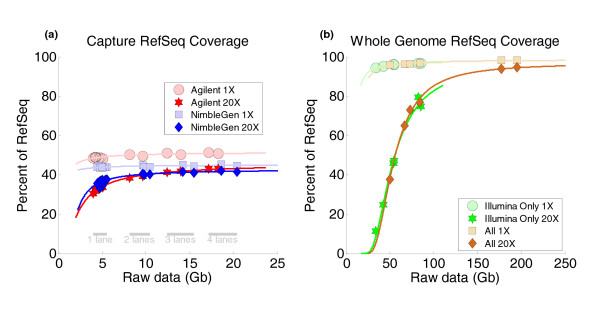
**Exome coverage, based on RefSeq sequences, was incomplete with exome capture but nearly complete with whole genome resequencing**. **(a) **Since the CCDS only includes very well annotated protein-coding regions, we assessed exome kit coverage of the more comprehensive RefSeq sequences, which include protein-coding exons, non-coding exons, 3' and 5' UTRs, and non-coding RNAs, and encompass 65,545,985 bp of genomic space. Coverage of RefSeq sequences by the exome kits was clearly incomplete, with at most 50% of RefSeq covered at 1× depth or greater. **(b) **In contrast, coverage of RefSeq by whole genome data from the trio pilot of the 1000 Genomes Project was nearly complete, with approximately 98% of RefSeq covered at 1× or greater and approximately 94% covered at 20× or greater from the more deeply sequenced daughter samples. This plot uses an identical format to the one used in Figure 2; see the caption of Figure 2 for detailed description.

It is important to mention that the amount of sequence data generated from each of the sequencing lanes used in this study was fairly consistent: 28 to 39 million pass-filter clusters per paired-end 76-cycle lane, corresponding to approximately 5 Gb of raw sequence data per lane. For clarity, we use one lane to represent one unit of raw data, except for data shown in Figures 1, 2, and 3, where the coverage of different targets is shown as a function of the amount of raw data, either in terms of lanes or in terms of bases. This demonstrates the variability in output from the lanes used in this study and allows, through interpolation, an estimation of the number of lanes necessary if different sequencing instruments or different read lengths are used.

We first calculated intended target coverage at selected sequencing depths. From a single lane of sequencing per capture, we obtained 61× to 93× mean depth across the NimbleGen target and 39× to 53× mean depth across the Agilent target (Figure 1a). When measured at 1× coverage, the NimbleGen platform captured 95.76 to 97.40% of its intended target, whereas the Agilent platform captured 96.47 to 96.60% of its intended target. The 1× coverage shows how much of the target can potentially be covered and, not surprisingly, we obtained similarly high coverage of the intended targets for each platform. However, we observed differences between the two kits when we measured coverage at read depths of 20×, which is a metric we use to support reliable variant detection. At 20× coverage, the NimbleGen kit covered 78.68 to 89.05% of its targets, whereas the Agilent kit performed less well, and covered 71.47 to 73.50% of its intended targets (Figure 1a). It should be noted that, in summary, these results also show that the commonly used metric of mean coverage depth has almost no value in capture experiments since the distribution of reads is uneven as a result of the capture.

Importantly, improved coverage was obtained with additional sequencing lanes, although the two platforms performed differently in terms of the extent and rate of improvement (Figure 1a). At 20× depth from multiple lanes of data, the NimbleGen platform produced a modest increase in breadth of coverage compared with one lane of data. However, the Agilent platform showed a more significant increase in breadth of coverage at 20× depth from multiple lanes of data. Thus, the NimbleGen kit was more effective at capture with less raw data input. The NimbleGen platform reached target coverage saturation with two lanes of data, whereas the Agilent platform required at least four lanes. This suggests that the Agilent kit provides less uniformity of capture across the target.

We next analyzed how well each product targeted the exons annotated in the CCDS. The approximately 27.5 Mb hg18 CCDS track is a highly curated representation of protein-coding exons whose annotations agree between various databases [12], and was the source of the protein coding regions targeted by the NimbleGen and Agilent capture platforms.

From one lane of data per sample, the NimbleGen platform covered 86.58 to 88.04% of the CCDS target at 1× depth, whereas the Agilent platform covered 95.94 to 96.11% of the CCDS target at 1× depth (Figure 1b). The two platforms performed as we had predicted from our theoretical calculations (see above). In contrast, at 20× depth NimbleGen covered 71.25 to 80.54% of CCDS while Agilent covered 72.06 to 73.82%. As mentioned above, with multiple lanes of data per sample, CCDS coverage at 20× improved for both platforms, while producing only a modest increase in CCDS coverage at 1×. Again, the increase at 20× was substantially larger for Agilent. For example, with four lanes of data, NimbleGen covered 85.81 to 85.98% of the target at 20× (approximately 10% more than the 20× coverage with one lane), while Agilent covered 90.16 to 90.59% (approximately 20% more than the 20× coverage with one lane). These results are consistent with our observation that the NimbleGen platform is more efficient at providing significant coverage of regions that it was designed to capture, though it targets a smaller percentage of the CCDS regions.

### Human exome coverage from solution exome capture versus whole genome sequencing

Given that a greater sequencing depth would be required in order to cover the CCDS to the same extent if the entire genome was sequenced, we wanted to determine the efficiency of exome capture and sequencing to that obtained with whole genome sequencing. To accomplish this, we used whole genome sequence data for the CEU and YRI trio samples, generated and made publically available by the 1000 Genomes Project [13].

The 1000 Genomes Project reported an average of 41.6× genome coverage for the trio pilot samples, although there was substantial variability among the coverage of the individual samples. The genomes of the daughter samples were covered at 63.3× (CEU daughter) and 65.2× (YRI daughter), while their parents were covered at 26.7×, 32.4×, 26.4×, and 34.7× (CEU mother, CEU father, YRI mother, and YRI father, respectively) [13]. When we measured the depth of coverage over the CCDS target, after downloading the alignment files and filtering for reads mapping to CCDS sequences with quality ≥ 30 [15], we observed a somewhat lower mean of 36.9× for the six individuals.

Although the variability of genome depth across the samples did not affect the CCDS coverage results at 1×, it had a major effect on the CCDS coverage at 20×. For example, while the YRI mother had a mean depth of 16.64× across CCDS, with 37.71% of CCDS covered at 20×, the YRI daughter had a mean depth of 65.15× across CCDS, with 94.76% of CCDS covered at 20×. The relationship between the mean depth and the percent covered at 1× and 20× is clearly demonstrated in Figure 2. Instead of plotting the actual mean depths of CCDS coverage obtained from the whole genome sequence data we analyzed, we extrapolated and plotted the amount of raw data that should be necessary to achieve such coverage depths. For the extrapolation we made two assumptions. First, we assumed that in order to get a certain mean depth across CCDS with whole genome sequencing, we would need to cover the whole genome at the same mean depth. Second, we optimistically assumed that in order to have the 3-Gb long human genome covered at a depth of D we would need three times D Gb of raw data (that is, we assumed that no data are wasted or non-specific in whole genome sequencing). We choose to use these two assumptions instead of plotting the specific raw data we downloaded from the 1000 Genomes Project because these data consist of predominantly 36-base reads with poor quality. With longer-cycle (for example, 100 or more) paired-end runs producing high quality sequence data, achieved routinely by us and others in the past year, our optimistic second assumption is only slightly violated. Having the x-axis of the plot in Figure 2 expressed in terms of raw data makes the relationship between raw data and target coverage in Figure 2 directly comparable to the plot in Figure 1b, which shows the extent of CCDS coverage obtained from using the NimbleGen or Agilent exome capture kits.

Whole genome sequencing at 20× genome depth covered more than 95% of the CCDS annotated exons (Figure 2). However, this required approximately 200 Gb of sequence, considering the results from the deeply covered daughters. This is in comparison to the roughly 90% coverage at 20× or greater of regions corresponding to the CCDS annotations by Agilent capture (or 85% coverage by NimbleGen) requiring only approximately 20 Gb of raw sequence (Figure 1b). It is possible that the newer sequencing chemistry used for the exome sequencing was partially responsible for this difference. However, it seems clear that even by conservative estimates exome sequencing is able to provide high coverage of target regions represented in the CCDS annotations 10 to 20 times as efficiently as whole genome sequencing, with the loss of 5 to 10% of those CCDS exons in comparison to whole genome sequencing.

### Capturing and sequencing regions not included in CCDS

The approximately 27.5 Mb hg18 CCDS track is a highly curated representation of protein-coding exons whose annotations agree between various databases [12], and the CCDS track was the source of the protein coding regions targeted by the NimbleGen and Agilent capture platforms. As described above, both reagents efficiently capture the vast majority of those exons.

The approximately 65.5 Mb hg18 RefSeq track, while also curated and non-redundant, is a much larger and less stringently annotated collection of gene models that includes protein coding exons (33.0 Mb), 5' (4.5 Mb) and 3' (24.1 Mb) UTRs, as well as non-coding RNAs (3.9 Mb) [8,9]. Not surprisingly, since the exome capture reagents are targeted against CCDS annotations, they did not cover approximately 6 Mb of potential protein coding regions as well as the 5' and 3' UTR regions (Figure 3a), resulting in at most approximately 50% of RefSeq annotations covered by the exome kits (Additional file 1). On the other hand, greater than 95% of RefSeq was covered from the whole genome data from any of the six trio samples, and greater than 98% of RefSeq was covered from the whole genome data from either of the more deeply sequenced daughter samples (Figure 3b; Additional file 1).

In addition to the global whole exome level, we looked at the coverage of individual genes. We considered two measures of gene coverage: (1) which genes and how much of each gene were targeted by a particular exome kit according to the intended target; and (2) the proportion of bases of each gene for which we were able to call genotypes (both measures were based on the coding regions of RefSeq). Surprisingly, quite a few medically important genes were not directly targeted by either the NimbleGen or the Agilent exome kits. Two examples of particular interest to us were *CACNA1C *(voltage-dependent L-type calcium channel subunit alpha-1C), which is one of the few bipolar disorder gene candidates, and *MLL2*, which is implicated in leukemia and encodes a histone methyltransferase. The reason these genes were not targeted was that neither of them were included in the CCDS annotations. Moreover, there was a large set of genes that, although targeted, were not covered sufficiently for genotype calls (for example, *APOE *(apolipoprotein E), *TGFB1 *(transforming growth factor beta 1), *AR *(androgen receptor), *NOS3 *(endothelial nitric oxide synthase)). This points to the limitations of using capture technology based solely on CCDS annotations. We provide a complete gene coverage report in Additional file 2. These limitations are important when considering the results of published exome sequencing projects, particularly negative results, since they may be caused by the exon of importance not being present in the CCDS annotations or by the important variant being non-coding.

### Factors that influence capture performance

The factors that influence all next generation sequencing results, whether from whole genome or hybrid selection, include sample quality, read length, and the nature of the reference genome. Although a powerful and cost- and time-effective tool, target capture carries additional inherent variables. In addition to the nature and restrictions of probe design [10,11], the success of target capture is particularly sensitive to sample library insert length and insert length distribution, the percent of sequence read bases that map to probe or target regions, the uniformity of target region coverage, and the extent of noise between capture data sets. These performance factors directly influence the theoretical coverage one may expect from the capture method and therefore the amount of raw sequence data that would be necessary for providing sufficient coverage of genomic regions of interest.

**Figure 4:**
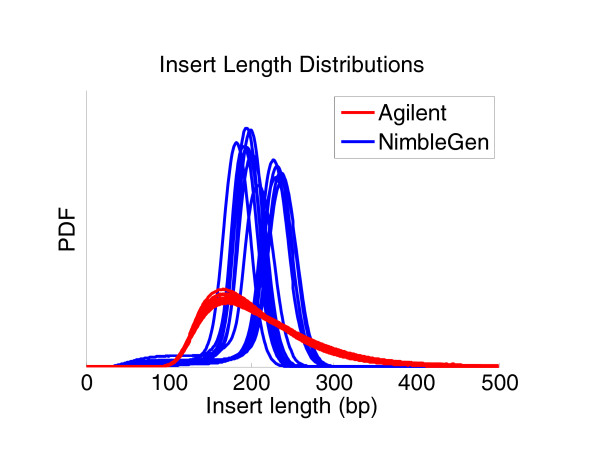
**Insert size distributions differed between the sample libraries prepared for the NimbleGen and Agilent exome capture kits**. Sample libraries were produced independently and were prepared according to the manufacturer's guidelines. The insert size distributions were generated based on properly mapped and paired reads determined by our capture analysis pipeline. The NimbleGen library preparation process involved agarose gel electrophoresis-based size selection, whereas the Agilent process involved a more relaxed, bead-based size selection using AMPure XP (Beckman Coulter Genomics). Bead-based size selection is useful for removing DNA fragments smaller than 100 bp but less effective than gel-based size selection in producing narrow size distributions. Yet, from a technical standpoint, the gel-based process is more susceptible to variability of mean insert size. The two different size selection processes are illustrated by our group of NimbleGen capture libraries and our group of Agilent capture libraries. PDF, probability distribution function.

Our analysis pipeline generates library insert size distribution plots based on alignment results. Since the NimbleGen and Agilent platforms utilized different sizing techniques in their standard sample library preparation workflows, the greatest difference in insert size distribution was observed between libraries prepared for different platforms (Figure 4). The NimbleGen workflow involved a standard agarose gel electrophoresis and excision-based method, whereas the Agilent workflow applied a more relaxed small-fragment exclusion technique involving AMPure XP beads (Beckman Coulter Genomics). Overall, there were tight and uniform insert size distributions for the NimbleGen capture libraries, ranging from 150 to 250 bp and peaking at 200 bp, whereas the insert size distributions for the Agilent libraries were broader, starting from approximately 100 bp and extending beyond 300 bp. Despite producing inserts that are more narrowly distributed, the process of gel-based size selection is more susceptible to variation inherent to the process of preparing electrophoresis gels and manually excising gel slices. The bead-based size selection process provides the benefit of less experiment-to-experiment variation.

One of the most important metrics for determining the efficiency of a capture experiment is the proportion of targeted DNA inserts that were specifically hybridized and recovered from the capture. Our analysis pipeline calculates enrichment scores based on the proportion of sequence bases that map specifically to target bases. With the NimbleGen platform 87.20 to 90.27% of read pairs that properly mapped to the genome were also mapped to probe regions, whereas with Agilent this metric was only 69.25 to 71.50%.

**Figure 5:**
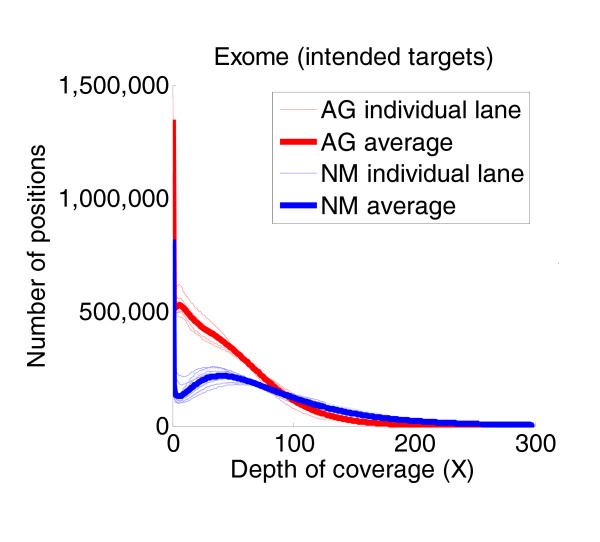
**Uniformity plots of exome capture data revealed fundamental differences in uniformity of target coverage between exome capture platforms**. The numbers of platform-specific target bases covered from 0× to 300× depth coverage are plotted for NimbleGen (NM) and Agilent (AG) exome captures. The NimbleGen exome data were more efficient at covering the majority of intended target bases, but the corresponding uniformity plots from these data revealed that there was also some over-sequencing of these positions, which thus broadened the coverage distribution for the NimbleGen targets. The Agilent exome data, however, showed significantly more target bases with no coverage or very poor coverage compared to the NimbleGen data, thus indicating that the Agilent data provided less uniform target coverage than the NimbleGen data. The lower uniformity of coverage produced from the Agilent captures results in the need to provide more raw sequence data in order to generate adequate coverage of targets. The Agilent platform was thus less efficient at target capture than the NimbleGen platform.

The more uniform the coverage across all targets, the less raw data are required to cover every target to a reasonable depth, thereby increasing the sequencing efficiency. The uniformity is represented by the distribution of the depths of coverage across the target. Figure 5 shows the depth distributions obtained with one lane from each exome capture and the average depth distributions obtained from the NimbleGen and Agilent captures. The two average distributions differed significantly, and neither displayed optimal coverage uniformity. A larger portion of the Agilent targets was insufficiently covered, whereas some of the NimbleGen targets were covered at higher depths than necessary.

**Figure 6:**
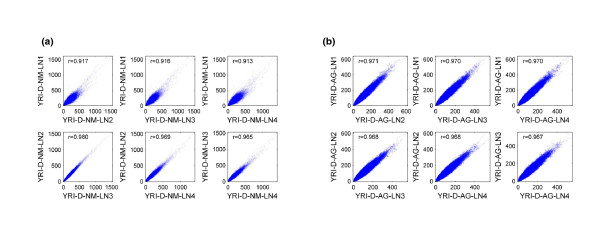
**Depth correlation plots prepared from exome capture data revealed that artificial background noise arising from the use of target capture kits might be problematic**. **(a) **Correlations of target base coverage depth between four independent NimbleGen captures with the daughter sample from the YRI trio (YRI-D-NM). Two different lots of NimbleGen exome probe libraries were used for this analysis, and correlation anomalies were only observed when comparing data between the two lots. YRI-D-NM-LN1 was captured with one lot and YRI-D-NM-LN2, YRI-D-NM-LN3, and YRI-D-NM-LN4 were captured with the other. **(b) **Correlations of target base coverage depth between four independent Agilent captures with the daughter sample from the YRI trio (YRI-D-AG). Only one lot of Agilent exome probe library was used for this analysis, and data between different captures consistently correlated well. AG, Agilent exome; D, YRI daughter; LN, lane; NM, NimbleGen exome; r, correlation coefficient.

Examining the results from multiple exome captures from the same source material allowed us to investigate experiment-to-experiment variation in the depth of coverage (Figure 6). Comparing the depth of target base coverage from a single replicate capture against any other replicate capture from the same individual, there was significant concordance for both the NimbleGen and Agilent exome platforms. Of note, inconsistencies were found between the NimbleGen captures, for which it appeared that captures performed with one lot of the exome kit produced slightly poorer correlations when compared to captures performed with a different lot. Although the use of different NimbleGen exome kit lots was not intentional, these results emphasize the necessity to consider potential differences between different probe lots if a given capture project will require the use of multiple lots for integrated analyses. All Agilent captures were performed with a single kit lot. Given the additional sample processing steps required for the hybrid capture workflow relative to whole genome resequencing, the consistency of the necessary reagents and procedures is an important factor that should be carefully monitored in order to minimize potential experimental artifacts.

### Genotyping sensitivity and accuracy of exome capture

It was previously reported that various genome capture methods, including array capture and solution capture, are capable of producing genotype data with high accuracies and low error rates [16]. These performance metrics are clearly important for properly evaluating targeted resequencing methods, which carry the caveat of generally requiring more sample handling and manipulation than whole genome resequencing. In addition, if the downstream goal of targeted resequencing is to identify sequence variants, one must consider the efficiency of exome capture for genotyping sensitivity and accuracy. Therefore, in addition to investigating the extent of the human exome that can be effectively captured in the context of exome coverage attained by whole genome sequencing, we further analyzed exome capture sequence data for these two parameters. We used the genotype caller implemented in the SAMtools package [17], and considered a genotype at a given position to be confidently called if the Mapping and Assembly with Quality (Maq) consensus genotype call [15] was ≥ 50 (10^-5 ^probability of being an incorrect genotype). Table 2 lists the percentage of the CCDS target for which genotypes were confidently called, and further describes the different types of variants that were called. There were more variants observed in the YRI sample than in the CEU sample, which is consistent with prior findings [18]. From this analysis it is also apparent that more data (for example, more sequencing lanes) leads to improved coverage and thus the ability to assign genotypes over a larger proportion of the region of interest. This trend is more pronounced with the Agilent exome data, which we believe to be due to factors that influence capture performance (see above). With NimbleGen exome captures, one lane of data provided enough coverage to support the assignment of genotypes to 85% of the CCDS target, and the data from four lanes provided a minor increase to 87%. With Agilent exome captures, the increase in coverage per amount of data was substantially larger: 86% of CCDS genotyped with one lane of data and 94% of CCDS genotyped with four lanes of data. While the Agilent kit provides the potential benefit of almost 10% more CCDS coverage for genotyping, it is important to note that this comes with the cost of requiring significantly more sequence data.

**Table 2:** Genotyping results obtained from exome capture data produced in this study

	Percentage	Variant	Heterozygous		Homozygous		Transition/transversion
				
Sample	called	number	**Syn**.	**Non-syn**.	**Syn**.	**Non-syn**.	ratio
CEU-D-NM-LN1	80.85	12,994	3,547	4,359	2,828	2,260	3.48
CEU-F-NM-LN1	79.68	12,501	3,413	4,182	2,726	2,180	3.45
CEU-M-NM-LN1	84.93	13,934	3,848	4,668	3,019	2,399	3.35
YRI-D-AG-LN1	86.38	17,214	5,161	6,082	3,266	2,705	3.44
YRI-D-AG-LN12	91.48	18,803	5,608	6,728	3,530	2,937	3.43
YRI-D-AG-LN123	93.33	19,468	5,804	6,974	3,665	3,025	3.41
YRI-D-AG-LN1234	94.17	19,719	5,869	7,061	3,708	3,081	3.41
YRI-D-AG-LN2	86.09	17,145	5,097	6,110	3,236	2,702	3.47
YRI-D-AG-LN3	86.04	17,161	5,127	6,091	3,238	2,705	3.47
YRI-D-AG-LN4	84.99	16,708	4,976	5,909	3,176	2,647	3.45
YRI-D-NM-LN1	84.27	17,146	5,014	6,273	3,272	2,587	3.47
YRI-D-NM-LN12	86.15	17,864	5,258	6,530	3,378	2,698	3.38
YRI-D-NM-LN123	86.71	18,081	5,328	6,608	3,405	2,740	3.35
YRI-D-NM-LN1234	87.01	18,208	5,376	6,642	3,426	2,764	3.33
YRI-D-NM-LN2	84.17	17,341	5,080	6,359	3,292	2,610	3.44
YRI-D-NM-LN3	84.06	17,328	5,101	6,336	3,289	2,602	3.40
YRI-D-NM-LN4	83.92	17,213	5,033	6,319	3,268	2,593	3.44
YRI-F-NM-LN1	85.26	17,389	5,006	6,217	3,446	2,720	3.38
YRI-F-NM-LN12	86.57	17,820	5,128	6,366	3,517	2,809	3.35
YRI-F-NM-LN2	84.62	17,294	4,966	6,194	3,419	2,715	3.41
YRI-M-AG-LN1	86.20	16,991	5,101	5,974	3,226	2,690	3.39
YRI-M-AG-LN12	90.97	18,452	5,523	6,539	3,501	2,889	3.36
YRI-M-AG-LN123	92.89	19,086	5,685	6,798	3,606	2,997	3.35
YRI-M-AG-LN1234	93.97	19,423	5,799	6,917	3,669	3,038	3.33
YRI-M-AG-LN2	83.48	16,095	4,859	5,619	3,059	2,558	3.37
YRI-M-AG-LN3	84.59	16,472	4,933	5,772	3,136	2,631	3.42
YRI-M-AG-LN4	85.95	16,832	5,032	5,897	3,215	2,688	3.38
YRI-M-NM-LN1	84.85	17,195	5,028	6,259	3,278	2,630	3.35
YRI-M-NM-LN12	86.33	17,742	5,219	6,458	3,358	2,707	3.29
YRI-M-NM-LN123	86.81	17,936	5,283	6,516	3,392	2,745	3.26
YRI-M-NM-LN1234	87.06	18,034	5,306	6,553	3,414	2,761	3.24
YRI-M-NM-LN2	84.52	17,222	5,043	6,271	3,285	2,623	3.35
YRI-M-NM-LN3	84.53	17,205	5,031	6,265	3,274	2,635	3.38
YRI-M-NM-LN4	84.40	17,197	5,045	6,252	3,268	2,632	3.36

Sequencing lanes were arbitrarily numbered from 1 through 4, irrespective of the actual lane on the Illumina flowcell the data were generated from. Lane data merges are represented by the digits after LN (lane), with LN1 representing the data from a single lane, LN12 representing the data from the two lanes LN1 and LN2, LN123 representing the data from the three lanes LN1, LN2, and LN3, and so forth. Genotypes were assigned based on consensus quality of 50 or above [15], and the proportion of the CCDS target bases genotyped using this filter is indicated for each dataset. The extent of CCDS coverage had a direct influence on the extent of genotyping we were able to carry out with each dataset. It is also apparent that increasing raw sequence data, obtained by lane merges in this study, results in increases in the number of genotypic variants detected. The YRI individuals were found to have a greater number of variants than the CEU individuals, which has also been determined in other studies [13,18]. Syn., synonymous.

To support our genotyping analyses and to examine the accuracy of our single nucleotide variant (SNV) calls, 'gold standard' genotype reference sets were prepared for each of the six CEU and YRI trio individuals based on the SNPs identified by the International HapMap Project (HapMap gold standard) and based on the genotype calls we independently produced, with parameters consistent with those used for our exome data, using the aligned sequence data from the trio pilot of 1000 Genomes Project (1000 Genomes Project gold standard).

Our HapMap gold standard is based on HapMap 3 [18], which we filtered for genotyped positions that are included in the CCDS. Approximately 43,000 CCDS-specific positions were genotyped in HapMap 3 for every individual. Of these, almost a quarter (11,000 positions) were variants and roughly two-thirds (6,700 positions) of these variants were heterozygous calls (Table 3). The HapMap project focuses on highly polymorphic positions by design, whereas the exome capture and resequencing method evaluated in this study aims to describe genotypes for all exonic positions, whether polymorphic, rare, or fixed, with the polymorphic genotypes being only a minority compared to genotypes that match the human reference. Thus, in order to have a more comprehensive gold standard, we used the whole genome sequence data generated from the two sets of trio samples by the 1000 Genomes Project, and collected all of the base positions that we were able to genotype with high confidence (minimum consensus quality of 100). As discussed above, the depth of whole genome coverage for the six trio samples varied substantially, from 20× to 60×. These differences in genome depth influenced the number of gold standard positions we were able to generate for each of the different samples. For example, the data from the mother of the YRI trio provided only 2.3 million confidently genotyped positions, while the data from the daughter of the YRI trio provided 25.8 million confidently genotyped positions. Only a small subset of the 1000 Genome Project standard positions had a genotype that was not homozygous for the allele in the reference genome (Table 2).

**Table 3:** Description of the HapMap and the 1000 Genomes Project gold standards used in this study

	HapMap			1000GP Whole Genome		
	
Sample	Positions	Variants	Heterozygous variants	Positions	Variants	Heterozygous variants
CEU-D	42,964	11,558	6,568	24,926,557	14,605	9,595
CEU-M	42,967	11,455	6,460	7,038,292	3,489	2,514
CEU-F	43,049	11,461	6,498	12,227,792	6,030	4,012
YRI-D	43,161	12,320	7,041	25,818,250	18,730	12,569
YRI-M	43,219	12,205	6,843	2,356,922	1,136	831
YRI-F	43,246	12,202	6,734	11,322,826	6,393	4,052

Both gold standards comprise a set of positions within CCDS for each of the six samples for which genotypes are given. We use the gold standards to compare the given genotypes to the genotype calls we make from our capture data over the same positions. The positions for the HapMap gold standard are taken to be the CCDS positions that have been successfully genotyped by the HapMap project using SNP arrays. The positions for the 1000 Genomes Project gold standard are the ones for which we were able to obtain high confidence (minimum consensus quality of 100) genotype calls based on the 1000 Genomes Project trio pilot sequence data. For both gold standards, the Positions column specifies the number of positions in the gold standard for each of the six samples, the Variants column specifies the number of gold standard genotypes that differ from the reference allele at the corresponding position, and the Heterozygous variants column specifies the number of heterozygous gold standard genotypes.

**Figure 7:**
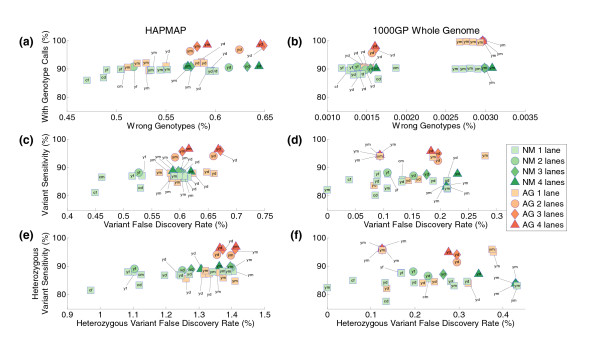
**Assessments of the genotyping performance of exome capture and resequencing over the CCDS target**. Exome capture sequence data were analyzed using our capture analysis pipeline (see Materials and methods; Figure 8), and genotype calls with consensus quality of at least 50 were used to determine the utility of solution exome capture for proper genotyping. These tests were performed with genotype gold standards prepared from the HapMap 3 panel and the trio pilot of 1000 Genomes Project (1000GP) for the two CEU and YRI trios used for this study (Table 3). In all panels, the color of the symbols designates the platform used, with green representing the NimbleGen platform (NM) and red representing the Agilent platform (AG). The label associated with the symbol identifies the sample using a two-letter code: the first letter identifies the trio (y for YRI and c for CEU) and the second letter identifies the family member (m for mother, f for father, and d for daughter). The shape of the symbols specifies the number of lanes of data used (rectangle for one lane, circle for two lanes, diamond for three lanes, and triangle for four lanes). **(a, b) **The y-axes show the percentage of the HapMap (a) and 1000 Genomes Project (b) gold standard positions that were successfully genotyped with a minimum consensus of 50; the x-axes show the percent of the called genotypes that disagree with the given gold standard genotypes. **(c, d) **Plots of sensitivity versus false discovery rates for the task of identifying variants: HapMap (c); 1000 Genomes Project (d). Sensitivity is defined as the percentage of positions with a variant genotype in the gold standard that have been called as variants from the exome capture data. The false discovery rate is defined as the percentage of variant calls from the exome capture data over the gold standard positions that do not have a variant genotype in the gold standard. **(e, f) **Plots of sensitivity versus false discovery rates for the task of identifying heterozygous variants: HapMap (e); 1000 Genomes Project (f).

We first assessed the accuracy of our CCDS genotype calls based on our exome capture data, which is a measure of whether our genotype calls (variant or reference) are consistent with a given gold standard. We found that we attained accuracies greater than 99% for each individual based on both types of our gold standards (Figure 7a, b). It is notable, however, that our accuracies were more than two orders of magnitude greater when we used the 1000 Genome Project gold standard (> 99.9965%) than when we used the HapMap gold standard (> 99.35%). We believe that this is due to variant genotypes being informatically harder to call with high confidence than reference genotypes, and that this is directly reflected by the variant-focused nature of our HapMap gold standard. Additionally, the 1000 Genomes Project sequence data that we used to generate our sequencing gold standard were obtained through next-generation sequencing, which is more consistent with our exome capture data than the data from the SNP arrays used for genotyping in the HapMap project.

We also tested the ability of our pipeline to identify positions with genotypes that differed (homozygous or heterozygous variation) from the human genome reference, and to specifically identify positions with heterozygous genotypes. For our analyses, we focused on the sensitivity of our method (the proportion of gold standard variants that were correctly called a variant from the captured data), and the false discovery rate of our method (the proportion of our variant calls at gold standard positions that were not in the list of variants within the gold standards). For both tests, we used the SNV calls generated from our exome captures and qualified them against both our HapMap and our 1000 Genomes Project gold standards (Figure 7c-f). For both our capture genotype calls and the two sets of gold standards we used, there is the possibility of missing one of the alleles of a heterozygous genotype and making an incorrect homozygous call (due to spurious or randomly biased coverage of one allele over the other), thus making the detection of heterozygous genotypes more challenging. Consistent with this challenge, we observed a larger proportion of false discoveries for heterozygous variants with respect to both gold standards. For example, up to 1.5% of our heterozygous calls were not in agreement with our HapMap gold standards. Consistent with our findings regarding the genotyping accuracy of our method, our error rates associated with correct variant identification were lower based on our 1000 Genome Project gold standards. On the other hand, we observed no differences in the genotyping sensitivity of our method based on the two types of gold standards. However, as reflected in our coverage results, we observed that the genotyping sensitivity associated with our Agilent exome captures improved with increasing amounts of sequence data. This was not necessarily the case for our NimbleGen exome captures since the coverage generated by these captures was less dependent on the data generated from multiple lanes of data. The high accuracy and high sensitivity of our exome captures are consistent with what was reported by Teer *et al. *[16], and support the utility of exome capture and resequencing when the entire genomic region of interest is adequately covered by the capture method.

## Discussion

Genome enrichment by hybridization techniques has shown rapid progress in its development and usage by the scientific community. The success of solution hybridization represents a transition for the capture methodology where the technique has become much more accessible for experimentation and more readily adaptable for high-throughput genetic studies. As with any experimental technique, there are both strengths and limitations, and it is important to understand these for accurate data interpretation. Herein we comprehensively identify important variables and critical performance liabilities and strengths for two solution exome capture products (Agilent and NimbleGen), and examine this with respect to whole genome resequencing. These analyses are crucial for the interpretation of exome capture projects, some involving hundreds or thousands of samples, that are in progress or have been completed using commercial exome kits.

Our results are consistent with the understanding that capture methodology is heavily design dependent [16]. Subsequent to these analyses, both NimbleGen and Agilent have released updated versions of their solution exome capture kits that are designed based on the latest assembly of the human genome reference, hg19 (GRCh37), and target both RefSeq (67.0 Mb) and CCDS (31.1 Mb) annotations. Looking forward, we computed hg19 CCDS and hg19 RefSeq coverage predictions based on the updated exome target files from NimbleGen and Agilent. The NimbleGen version 2 exome targets 9.8 Mb more genomic space (36.0 Mb total) than version 1, and we predict version 2 would provide 99.2% coverage of CCDS (approximately 10% more than version 1). However, the extent of version 2 target base overlap with RefSeq suggests that only 49.6% of RefSeq would be covered. The development of exome capture by Agilent has thus far produced two newer exome kits, one that targets 8.7 Mb more genomic space (46.2 Mb total; version 2) than version 1, and another that targets 13.9 Mb more genomic space (51.5 Mb total; version 3) than version 1. We predict that the newer Agilent kits should provide 96.3 to 98.1% of CCDS and 49.3 to 51.8% of RefSeq. While these kits will be invaluable for many researchers, others who are interested in regions not targeted in these kits will need to opt for ordering custom capture designs.

Beyond investigating the coverage limitations of exome capture kits, we determined that the high confidence genotypic information produced by exome capture and resequencing provides accuracies greater than 99.35%, sensitivities up to 97%, and false discovery rates up to 0.67% for all variants and up to approximately 1.5% for heterozygous variants (Figure 7). In this regard, the results of our assessment of exome capture genotyping accuracy and power are consistent with what has been previously reported [16].

In addition to investigating the performance of exome resequencing relative to whole genome sequencing and array-based genotyping (SNP arrays), we studied the consistency of our data by correlating the sequence coverage depths between independent replicate captures for a given DNA sample. We found significant correlations for both the NimbleGen and the Agilent exome capture platforms, with possible variations between different capture probe lots influencing the strength of correlations between captures (Figure 6). The extent of noise produced by the hybrid capture process is a distinctive parameter that does not influence whole genome resequencing. Alternatively, however, producing adequate whole genome coverage currently requires more extensive sequencing than producing adequate exome coverage, which introduces variables that can be challenging to control (for example, multiple sequencing runs, necessity for longer read lengths of high quality). Overall, the findings from this study underscore the importance of sequence capture uniformity and capture probe performance, which directly influence the amount of raw sequence data necessary to produce adequate target coverage for downstream data analysis.

Our results clearly show both the value of exome capture approaches and their relative limitations in capturing salient variation in the human genome. It is important to recognize that critically relevant, disease-associated variants are not found only in coding exons [19-21]. Whole genome sequencing offers the least biased and most comprehensive method of studying the human exome, and additionally provides one with the option to study potentially relevant variants in the non-coding regions of the human genome or coding regions that had not initially been annotated as such. Whole genome sequencing is also significantly more suitable for studies designed to investigate structural variants such as copy number variants, translocations, and fusion events.

For exome resequencing projects, the drawback of having to handle the much larger data sets presented by whole genome sequencing might be reasonably offset by a need to produce comprehensive data, and by performing family based analyses as an efficient means of filtering data sets for finding genetic candidates of highest priority or interest. The argument for performing whole genome resequencing in situations requiring, at the minimum, true whole exome coverage becomes stronger with the rapidly dropping cost of massively parallel sequencing using newer sequencers such as the Illumina HiSeq 2000 instrument, juxtaposed with the cost of performing hybridization-based enrichment and resequencing.

## Conclusions

We show relatively small but consistent differences between exome and genome sequencing in terms of providing sequence coverage of the regions of the genome represented by CCDS. Moreover, significant genes are not present in the CCDS annotations and hence not targeted by exome sequencing. This, combined with the general absence of non-coding exons in the regions annotated by CCDS, is apparent in our data, which shows only about 48% of the more expansive RefSeq annotated sequences are effectively sequenced by exome capture. While not surprising, since the regions were not targeted for capture, such data are important in interpreting published exome capture results, particularly negative results. Our data also underscore the need for critical evaluation of positive results from exome capture kits, since they cannot provide the 'completeness' of analysis that genome sequencing can provide.

One area where targeted sequencing will likely see even greater value is in the custom capture of much smaller regions of the genome in a highly multiplexed fashion, for which the difference in cost compared to whole genome sequencing would be too great to support a workflow that does not involve target capture. Ongoing large sample size exome resequencing projects, as well as various whole genome resequencing projects, will identify substantial numbers of potential candidate genes for a range of diseases and other phenotypes. Being able to efficiently direct the capability of next-generation sequencing instruments towards highly multiplexed resequencing of relatively small numbers of genes in large numbers of patients and controls is currently an unmet need that could potentially be addressed by hybridization-based target enrichment.

## Materials and methods

### DNA samples and publicly available data used for this study

Purified genomic DNA from cell lines of the CEU family trio individuals NA12892, NA12891, and NA12878 and YRI family trio individuals NA19238, NA19239, and NA19240, maintained at Coriell Cell Repositories in Coriell Institute for Medical Research (Camden, NJ, USA), was used for exome captures. The publicly released whole genome alignment and filtered sequence files from the high coverage trio pilot of the 1000 Genomes Project were downloaded from the NCBI FTP site [22]. The alignment files utilized were downloaded from the pilot_data directory of the FTP site, and the filtered sequence files were downloaded from the data directory of the FTP site. The genotyping data used as 'gold standards' for the six trio individuals were obtained from the International HapMap Project FTP site [23].

### Targets and gene annotations

For the CCDS annotations, CCDS version 20090327 was downloaded from the NCBI FTP site [12,24]. For RefSeq, the NCBI36.1/hg18 associated gene name and gene prediction (refFlat) and extended gene prediction (refGene) tables from the University of California, Santa Cruz (UCSC) Table Browser database on 7 September 2010 were downloaded [25,26]. The intended targets for NimbleGen and Agilent were provided by the two companies and were downloaded from their respective websites.

### Sample library preparation and whole exome solution captures

The CEU and YRI DNA samples were directly processed into Illumina sequencing compatible libraries (pre-capture) prior to exome capture. The DNA modification enzymes and reaction reagents necessary for the Illumina library preparation procedure were individually purchased from New England Biolabs (Ipswich, MA, USA) or Roche Applied Science (Indianapolis, IN, USA). All necessary oligos for Illumina library preparation or exome capture were purchased from Integrated DNA Technologies (Coralville, IO, USA).

For each exome capture platform, one to four independently prepared pre-capture libraries were generated from each DNA sample, for one capture or multiple captures, respectively, with a given sample. The pre-capture libraries were prepared according to the manufacturer's guidelines that accompanied the SeqCap EZ Exome Library SR (Roche NimbleGen, Madison, WI, USA) or the SureSelect Human All Exon Kit (Agilent Technologies, Santa Clara, CA, USA). Pre-capture libraries that were intended for NimbleGen exome captures were size-selected for approximately 290 bp library fragment size (including the Illumina adapter sequences on each end of a library fragment), using 2% Certified Low Range Ultra Agarose (Bio-Rad Laboratories, Hercules, CA, USA) in 1× TAE (40 mM Tris acetate, pH 8.0; 1 mM ethylenediamine tetraacetic acid) containing 0.5 μg/ml ethidium bromide, consistent with the user's guide accompanying the NimbleGen exome capture product and with other sequence capture procedures [27]. Pre-capture libraries that were intended for Agilent exome captures were broadly size-selected for the exclusion of DNA fragments less than approximately 150 bp, using AMPure XP (Beckman Coulter Genomics, Brea, CA, USA) according to the Agilent SureSelect Human All Exon Kit user's guide. Our NimbleGen and Agilent exome solution captures were carried out according to the manufacturer's guidelines, and post-capture library amplifications and quality assessments were also performed according to the manufacturer's guidelines.

### Illumina DNA sequencing of exome captures

Illumina (San Diego, CA, USA) sequencing of exome captures was performed on site, at Cold Spring Harbor Laboratory, using constantly maintained Genome Analyzer_IIx _instruments with paired-end modules. Each exome capture was individually sequenced in one lane of a Genome Analyzer_IIx _flowcell using paired-end 76-cycle sequencing chemistry. Collectively, the exome capture data were obtained from four separate Genome Analyzer_IIx _runs. Each exome capture lane generated 268,972 to 367,692 clusters per tile (raw), with 82.45 to 91.89% of the clusters passing the Illumina data quality filter. These exome capture sequence data have been deposited into the National Center for Biotechnology Information (NCBI) Sequence Read Archive [28].

### Initial sequence data analysis

Sequencing images that were generated on Genome Analyzer_IIx _instruments were processed and base calls and quality scores were generated on the fly using the Illumina Real Time Analysis software (RTA v1.8). The processed signal intensity files, base calls and quality scores were then transferred to a shared 2,000 core IBM blade cluster running Linux or to a dedicated 96 core Sun cluster running Linux for further analysis. The Offline Basecaller (v1.8) was used to convert the binary base call files to text format. The Illumina CASAVA pipeline (v1.6 or v1.7) was then used to determine initial genome alignment statistics for the sequence data. These versions of RTA and CASAVA allow images with a high density of clusters to be analyzed (in the range of 35 to 38 million clusters per lane), thereby providing greater data output with 70 to 80% of the sequences passing the standard quality filter. The GERALD module included in CASAVA provides the run summary and output statistics along with graphical data quality files.

### Capture data analysis pipeline

The main goal of our analysis pipeline is to reliably identify SNVs in the target regions of individual samples; a secondary goal is to produce detailed reports that can be used to monitor the performance of the sequencing experiments and to allow us to compare different sequencing strategies. We developed our pipeline around the *de facto *standard format SAM using the freely available tools BWA [14] and SAMtools [17]. We used Makefiles [29] to integrate the different steps and we used the qmake tool from the Sun Grid Engine platform to execute the pipeline on the large computational cluster BlueHelix at Cold Spring Harbor Laboratory.

**Figure 8:**
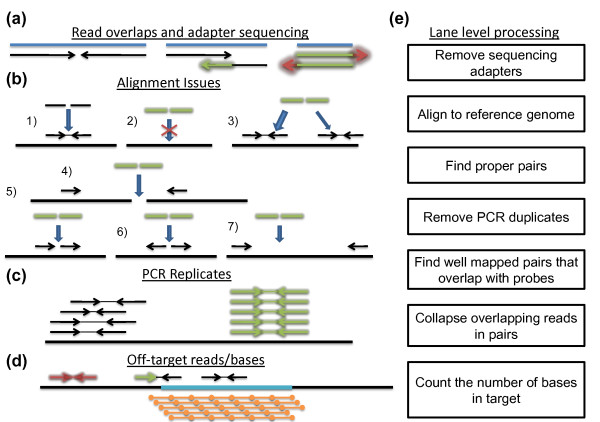
**Description of the lane-level processing of our analysis pipeline**. **(a-d) **The issues that our lane-level processing addresses. (a) Insert length-related complications. (b) The various ways a pair of reads can align, with 1) showing a proper-pair alignment. (c) How PCR duplicates look after alignment. (d) A cartoon of off-target reads and off-target bases of on-target reads. **(e) **The steps we take to address the issues demonstrated in (a-d). See the Materials and methods section for detailed descriptions.

An ideal capturing technique would ensure that all the bases produced by the sequencing machine would be aligned confidently on the target of interest, that the target would be covered uniformly, and that each base would provide an independent observation of the underlying genotype. This ideal cannot be achieved due to many factors of the sequencing strategy and the structure of the human genome. Figure 8 demonstrates some of the issues that arise and that are addressed in our analysis pipeline.

Figure 8a addresses the relationship between the sequenced insert length (insert here refers to the DNA molecule before ligating the sequencing and PCR primers) and the chosen read length. The expectation is that the insert is longer than the doubled read length and thus the paired reads from the ends of the insert would sequence different non-overlapping bases (Figure 8a, left). In reality, the insert lengths cannot be tightly controlled and a substantial proportion of the sequenced inserts might have lengths shorter than the doubled read length. In the data presented here, we used paired-end 76-cycle runs and from Figure 4 it is apparent that there were a number of inserts shorter than 152 bp. For shorter inserts, the ends of the two paired reads sequence the same nucleotide and for those the assumption of independent genotype observation is broken (Figure 8a, middle). In more extreme cases, the insert length is shorter than the length of a single read, and that leads not only to complete overlap of the two reads but also to the sequencing of the ligated adapters (Figure 8a, right). If not removed, the presence of these non-human bases interferes with the proper alignment of sequence reads.

When aligning a pair of reads we hope to find only one locus in the reference genome for which the two reads align close to each other in a way consistent with them being sequenced from the two ends of a short DNA insert (Figure 8b1). A pair that is aligned in this way is a 'proper pair'. (For Illumina pair-end sequencing a proper pair alignment implies that the read that aligns closer to the 5' of the reference chromosome is aligned on the forward strand and the pair closer to the 3' end is aligned on the reverse strand with respect the reference.) There are multiple ways for a pair to not be a proper pair. First, for some pairs there is no suitable locus in the reference genome (Figure 8b2). Second, there might be multiple candidate loci in the reference genome for a given pair (with identical or similar alignment scores; Figure 8b3). Third, the two reads can align on different chromosomes (Figure 8b4), align on the same chromosome in a wrong orientation (Figure 8b5 and 8b6), or align on the same chromosome far away from each other (Figure 8b7). Improper pairs can be caused by incorrect reference genome, by structural variants in the sample, or by a large number of sequencing or sample preparation protocol artifacts. Given that the focus of the pipeline is on SNVs in coding regions, we choose to analyze only proper pairs.

Several steps in the sample preparation and capture protocols require PCR amplification. As a consequence, a certain proportion of the original DNA inserts will be sequenced multiple times. One of the main benefits of paired-end sequencing is that it allows for a reliable identification of the identical copies based on their alignment coordinates. It is unlikely that two independent DNA inserts would have exactly the same genomic coordinates (both at the beginning and at the end) and if we do observe two or more read pairs aligning at the same coordinates, we can conclude that they are PCR copies of the same original insert (Figure 8c, right). Such redundant sequencing does not contribute independent observations of the underlying bases and, therefore, are removed prior to the SNV calling step.

A capture/enrichment strategy aims at sequencing DNA inserts that overlap the target of interest. The hybridization-based capture approaches achieve that by designing probes within or next to the target of interest. After the identification of the proper pairs we can easily identify the ones that have been specifically hybridized by searching for pairs that are aligned at a locus overlapping the designed probes (Figure 8d). The proportion of off-probe pairs is the most important measure of capture performance. In addition, not all the bases of the on-target proper pairs fall within the target of interest. The bases outside of the target cannot contribute to the SNV calls. The proportion of bases of the on-target proper pairs that fall outside the target is another measure of performance; it depends on probe design strategy and on the insert length distribution. For whole exome sequencing with an average exon length of about 150 bp, longer inserts (for example, longer than 200 bp) are not desirable.

The pipeline is split into lane-level processing and sample-level processing. The lane-level processing has seven steps.

Step 1 is removing sequencing adapters (Figure 8a, right). This step is implemented with our custom script that works by aligning the two reads of each pair against each other after reverse-complementing one of them while aligning the flanking sequence to the Illumina standard adapters.

Step 2 is aligning. For this we use BWA [14] in paired-end mode (aln and sampe commands) and with default parameters. For 76-base long reads, the default BWA parameters allow four differences (single nucleotide or an indel) between the read and the alignment reference locus. The default parameters also require BWA to report no more than one alignment location of a read with multiple possible locations (Figure 8b3). The mapping quality, defined as *q_m _*= -10 log_10_*P*, where *P *is the probability that the location provided is incorrect, produced by BWA reflects the degree of ambiguity. A mapping quality of 0 indicates that there are two or more equally good candidate locations in the reference genome. The maximum mapping quality reported by BWA is 60. In paired-end mode BWA reports two potentially different mapping qualities for the two reads of a pair. We assigned the minimum of the two mapping qualities as the mapping quality for the pair as a whole.

Step 3 is finding proper pairs. This is accomplished with a custom script that analyzes the FLAG field in the SAM file alignment records [17].

Step 4 is removing PCR duplicates. This step addresses the issue demonstrated in Figure 8c. The step is implemented with the SAMtools rmdup command [17].

Step 5 is finding well mapped read pairs that overlap with probes. This step uses a custom script that implements two filters simultaneously: exclusion of all read bases that do not map to exome capture probe regions (we require an overlap of at least 20 bases between a read and a probe region) and removal of proper read pairs with suboptimal mapping quality. We chose to use only pairs aligned with the maximum mapping quality of 60.

Step 6 is collapsing overlapping bases in read pairs. This step addresses the issue demonstrated in Figure 8a (middle). The two reads of a given pair with overlapping bases are shortened until the overlap is eliminated. The base quality scores are subsequently updated to increase certainty if the two reads agree at a given position or to decrease certainty in the case of disagreement. This step also removes all reads determined to contain insertion or deletion mutations.

Step 7 is counting and reporting the number of bases that fall within target regions.

In the sample-level processing there are three steps. In step 1 the data generated from different lanes containing the same sample are merged together (SAMtools merge command). In step 2 consensus genotypes are called using the SAMtools Maq-based model (pileup command with -A option). In step 3 the confident genotypes are filtered for those with genotype, or consensus, quality ≥ 50.

## Abbreviations

bp: base pair; BWA: Burrows-Wheeler Aligner software; CCDS: Consensus Coding Sequences; CEU: Utah residents with ancestry from northern and western Europe; Gb: gigabase; RefSeq: The Reference Sequence collection; GRCh37: Genome Reference Consortium human genome reference sequence assembly: build 37; Mb: megabase; NCBI: National Center for Biotechnology Information; PCR: polymerase chain reaction; SNP: single nucleotide polymorphism; SNV: single nucleotide variant; UCSC: University of California: Santa Cruz; UTR: untranslated region; YRI: Yoruba in Ibadan, Nigeria.

## Competing interests

The authors declare that they have no competing interests.

## Authors' contributions

JSP and II led the design and comparative analyses of the study and drafted the manuscript. IG performed the sample preparations and sequence captures. MSS contributed key insights to the formulation of the capture analysis pipeline and edited the manuscript. MK established an Illumina analysis pipeline for the initial assessment of newly generated sequence data. WRM conceived of the study and helped draft the manuscript. All authors read and approved the final manuscript.

## Supplementary Material

Additional file 1 **Full bioinformatic capture analysis report**. A full lane-level and sample-level report from our capture sequence data analysis pipeline. This report contains all of the statistics our standard pipeline generates. Three distinct sets of sample-level statistics are provided for the specific analyses we performed using various genomic targets of interest. Click here for file

Additional file 2 **Gene-specific coding region coverage from exome capture**. A table listing all UCSC hg18 RefSeq track genes, their predicted capture coverage (percent) based on capture kit intended targets, and their actual capture coverage (percent) from our data. GeneLength depicts the combined length of the coding regions of each gene, in bases. Click here for file
